# Computer simulations on oxidative stress-induced reactions in SARS-CoV-2 spike glycoprotein: a multi-scale approach

**DOI:** 10.1007/s11030-021-10373-6

**Published:** 2022-02-18

**Authors:** Oscar Bertran, Didac Martí, Juan Torras, Pau Turon, Carlos Alemán

**Affiliations:** 1 Departament de Física EETAC, Universitat Politècnica de Catalunya, c/Esteve Terrades, 7, 08860 Castelldefels, Spain; 2 Departament d’Enginyeria Química (DEQ) and Barcelona Research Center in Multiscale Science and Engineering, EEBE, Universitat Politècnica de Catalunya (UPC), C/Eduard Maristany 10-14, 08019 Barcelona, Spain; 3 B. Braun Surgical, S.A.U. Carretera de Terrasa 121, 08191 Rubí, Barcelona, Spain; 4 Institute for Bioengineering of Catalonia (IBEC), The Barcelona Institute of Science and Technology, Baldiri Reixac 10-12, 08028 Barcelona, Spain

**Keywords:** Hydrogen abstraction, Isoleucine, Molecular dynamics, Reactive oxygen species, Receptor binding domain, Spike protein

## Abstract

**Abstract:**

Oxidative stress, which occurs when an organism is exposed to an adverse stimulus that results in a misbalance of antioxidant and pro-oxidants species, is the common denominator of diseases considered as a risk factor for SARS-CoV-2 lethality. Indeed, reactive oxygen species caused by oxidative stress have been related to many virus pathogenicity. In this work, simulations have been performed on the receptor binding domain of SARS-CoV-2 spike glycoprotein to study what residues are more susceptible to be attacked by ·OH, which is one of the most reactive radicals associated to oxidative stress. The results indicate that isoleucine (ILE) probably plays a crucial role in modification processes driven by radicals. Accordingly, QM/MM-MD simulations have been conducted to study both the ·OH-mediated hydrogen abstraction of ILE residues and the induced modification of the resulting ILE radical through hydroxylation or nitrosylation reactions. All in all, in silico studies show the importance of the chemical environment triggered by oxidative stress on the modifications of the virus, which is expected to help for foreseeing the identification or development of antioxidants as therapeutic drugs.

**Graphic abstract:**

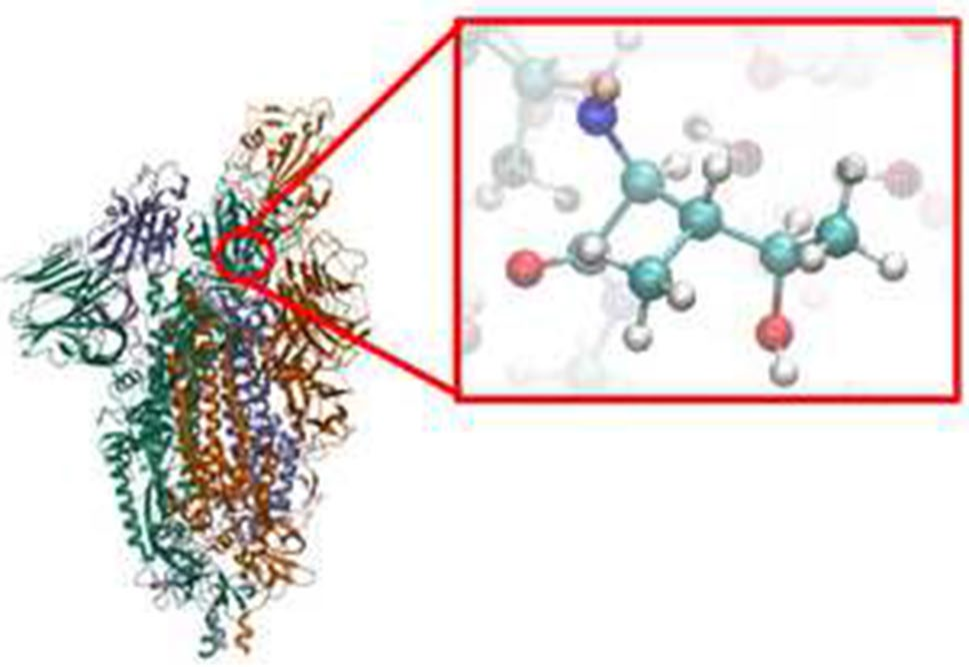

## Introduction

Oxidative stress occurs when an organism is exposed to an adverse stimulus that results in a misbalance of antioxidant and pro-oxidant species [1]. Accumulated research demonstrates that oxidative stress is a relevant factor associated with infectious diseases, such as Hepatitis B and C, Herpes Simplex, Human Immunodeficiency and Influenza viruses [2–4]. Furthermore, the spike protein of SARS-CoV-2 has been found to noticeably increase the levels of reactive oxygen species (ROS) [5, 6]. Oxidative stress is a common denominator of diseases considered as a risk factor for SARS-CoV-2 lethality (i.e., cancer, hypertension, vascular diseases and diabetes) [7, 8]. Some authors have related ROS with virus pathogenicity [9–11] considering the release of ROS and pro-oxidant cytokines as a result of virus activation of phagocytic cells, interfering in the balance between antioxidants and pro-oxidants of host cells [12]. Indeed, recent studies have evidenced relationships between oxidative stress and SARS CoV-2 infection, suggesting that ROS could be critical for disease progression [13–16].

Among ROS, hydroxyl radical (·OH) deserves special attention since it is one of the most reactive radicals, exhibiting a very short half-life (~ 1 ns at 37 ℃) and extremely high reaction rate constants [17]. ROS are able to modify molecules in their close environment and lead to diseases related to cell injury when a misbalance occurs (e.g., pneumonia, nervous system diseases and leukemia) [18–21].

We focus the present work on the direct interaction between the ·OH specie and the homo-trimeric spike protein of SARS-CoV-2 in order to elucidate how the glycoprotein will be affected by the radical. The knowledge of such interaction could provide additional understanding about the virus behavior in physiological environments that might result in functional changes. Furthermore, it aims to contribute to the development of antioxidants as therapeutic drugs to minimize such risk.

We focus on the present work on the interaction between the ·OH species and the homo-trimeric spike protein of SARS-CoV-2 in order to elucidate how the glycoprotein will be affected by the radical. The knowledge of such interaction is expected to allow for a better understanding about the virus behavior in physiological environments that might result in functional changes. Furthermore, it aims to contribute to the development of antioxidants as therapeutic drugs to minimize such risk.

## Methods

### Construction of the spike molecular model

The cryoelectron microscopy structure of the homo-trimeric spike glycoprotein of SARS-CoV-2 (PDBid: 6vxx), which solved at a resolution of 2.80 Å [22], was taken from the Protein Data Bank and used to prepare the initial model. The missing residues (i.e., 44–55, 88, 89, 118–139, 147–159, 217–236, 417–429, 445–462, 476, 595–614, 651–653, 802–828, 1162–1175, 1236–1258, 1268–1280, 1292–1294, 1307- 1309, 1338–1357, 1550–1556, 1562–1585, 1610–1616, 1716–1735, 1772–1783, 1923–1948, 2283–2296, 2360–2380, 2389–2401, 2459–2479, 2661–2263, 2671–2677, 2687–2706, 2837–2856, 2893–2905 and 3044–3071) were incorporated using the Modeler algorithm [23] implemented in the UCSF Chimera program [24] and the Z-DOPE (Discrete Optimized Protein Energy), statistical potential based for the choice of best model (i.e., that with the lowest Z-DOPE) for each conformational state, among the generated ones.

The homo-trimeric protein model with 9 Na^+^ to neutralize the charge was then submerged in a previously equilibrated water box of 19 × 20 × 22 nm^3^. Any water molecule that overlapped with any of the atoms belonging to the protein spike model was removed. After that, 440 randomly selected water molecules were replaced by 440 ·OH radicals. With the incorporation of an excessive amount of radicals, it was sought to increase the probability of contact within a limited time trajectory. Finally, the system contained a total of 212,198 water molecules. The model was completed by inserting randomly 440 Na^+^ and 440 Cl^–^ ions until reaching a concentration to reproduce physiological conditions. Then, the model was processed with the LEaP program [25] to add hydrogen atoms to the protein and to generate Amber topology files and coordinates files. Accordingly, the model presented 691,739 explicit atoms.

### Force field

All simulations were performed using the Amber 18 simulation suite [26]. Protein atoms were modeled using the Amber ff14SB force field [27], the glycan atoms included in the cryo-EM coordinates were modeled using the Glycam06 force field [28], and water atoms were modeled using the TIP3P force field [29]. This combination of force field parameters was found to be appropriate for the MD modeling of the spike glycoprotein of SARS-CoV-2 [30]. The geometrical and nonbonding parameters for the hydroxyl radical (·OH) were taken from Roeselová et al*.* [31]. In brief, geometrical parameters were taken from geometry optimizations at the MP2/aug-cc-pVTZ, while electrostatic atomic charges (–0.4 and + 0.4 for oxygen and hydrogen atoms, respectively) were obtained at the MP2/6-31G level. The van der Waals parameters (i.e., the atomic radius, R, and the hardness, ε) for the oxygen atom were R = 1.798 Å and ε = 0.156 kcal/mol, while they were set to zero for the hydrogen atom. Finally, the ·O–H stretching force constant was derived from the experimental harmonic frequency, 3738 cm^−1^ [32], as *k*_*s*_ = 1115.4 kcal/(mol·Å^2^).

### Computational details for classical MD simulations

Equilibration calculations were started by relaxing the protein regions filled with the UCSF Chimera program [24], which was achieved by applying the limited-memory Broyden–Fletcher–Goldfarb–Shanno quasi-Newton algorithm methodology to the added residues meanwhile the rest of the system was kept frozen. Next, the whole system was submitted to 2500 steps of full conjugate gradient minimization to relax conformational and structural tensions.

The Langevin dynamics method [33] was used to heat the system and to rapidly equilibrate its pressure and temperature. The relaxation times used for the coupling were 10 ps for both temperature and pressure. The temperature was increased from 0 to 500 K using 20 ps simulation in the NVT ensemble, keeping the frozen the atoms of the spike, maintaining the pressure at 1.034 bar and using an integration time step of 2 fs. After this, the temperature was decreased to 310 K using 0.2 ns of NVT MD simulation at 1.034 bar. In the latter process, the spike protein was still frozen. Then, 2 ns in the NPT ensemble was conducted at 310 K to relax the whole system, including the protein, and the density (integration step: 2 fs; pressure:1.034 bar). The last snapshot of this relaxation was used as starting point of the 250 ns NPT production trajectory at 310 K (integration step: 2 fs; pressure: 1.034 bar). Snapshots were stored every 10 ps.

Nonbonding pairs list was updated every 12 steps. Periodic boundary conditions were applied using the nearest image convention and the atom pair cutoff distance used to compute the van der Waals interactions was set at 10.0 Å. Beyond the cutoff distance, electrostatic interactions were calculated by using particle mesh of Ewald [34].

### Computational details for hybrid QM/MM-MD simulations

Hybrid quantum mechanics/molecular mechanics (QM/MM)–MD simulations were performed considering a sub-system containing the receptor binding domain (RBD) of SARS-CoV-2 in physiological conditions. In hybrid QM/MM-MD simulations the atomic motions are handled by MD, while energies and forces are calculated by dividing the system into two different parts. The reacting radical (·OH or ··NO) and ILE472 residue are treated at the quantum mechanical (QM) level while molecular mechanics (MM) are applied to the rest of the system (all the residues included in the RBD of SARS-CoV-2 with the exception of ILE472 residue and all the ions and water molecules) by using the classical potential energy function included in Amber program [26] and the set of force field parameters described above for classical MD simulation.

A total of 40 QM/MM-MD trajectories were conducted to study the ·H abstraction from the ILE472 residue. Previously to the production of the QM/MM-MD trajectories, selected snapshots taken from the classical MD simulation were modified by approaching the selected ·OH radical, which was at a distance < 4 Å from the reacting ILE, to a distance *R* (with *R* = 1.4 or 1.7 Å) of the ILE472 hydrogen atom chosen as reaction site. A total of 10 and 30 QM/MM-MD trajectories were conducted for *R* = 1.4 or 1.7 Å, respectively. After minimization at the MM level, these modified systems were heated up to 310 K and, finally, equilibrated by classical MD using a NPT ensemble for 1 ns (2 fs time steps). In both thermalization and equilibrations steps the distance *R* between the oxygen atom of the ·OH radical and the ILE472 reacting site was restrained by a force constant of 10 kcal/mol·Å^2^, whereas the rest of conditions were identical to those described above.

On the other hand, QM/MM-MD trajectories for the hydroxylation and nitrosylation of the ·ILE472 (the radical ILE residue obtained by ·H abstraction) were performed using a similar procedure. In this case ·OH and ^·^NO radicals were placed at distance *R* = 1.6 or 2.0 Å from the ·ILE472 reaction site. A total of 8 QM/MM-MD runs (4 for hydroxylation and 4 for nitrosylation), which were 0.2 ps long each, were conducted.

Hybrid QM/MM-MD simulations were run using PUPIL interface [35, 36], which allows to link, among others, QM calculations from NWChem [37] program with MD simulations from Amber [18, 26] program. The starting structures used for the QM/MM-MD simulations through the NWChem-PUPIL-Amber interface were extracted from the last classical MD equilibration snapshot. Subsequently, the reacting ILE residue with the closest neighbor atom of the adjacent residues and the selected ·OH radical was changed to a QM description, while the rest of the system remained within the MM framework. Thus, all atoms in the QM region were described by combining M06-2X functional [38, 39], which is a meta-generalized gradient DFT especially developed for thermochemical kinetics studies, with the 6–31 + G(d) basis set. After that, the systems were allowed to relax for 0.1 ps (0.5 fs time step) restricting the distance *R* between the oxygen atom of the radical and the ILE472 or ·ILE472 reacting site and using a constant pressure simulation with the same parameters previously used for fully classical MD simulations. Periodic boundary conditions were applied in the preparation of the NWChem input so as to wrap neighboring point charges around the quantum region. The, production QM/MM-MD trajectories (0.4 ps for ·H abstraction and 0.2 ps for hydroxylation and nitrosylation) were run and the coordinates were saved every 10 fs for subsequent analyses.

## Results and discussion

**Fig. 1:**
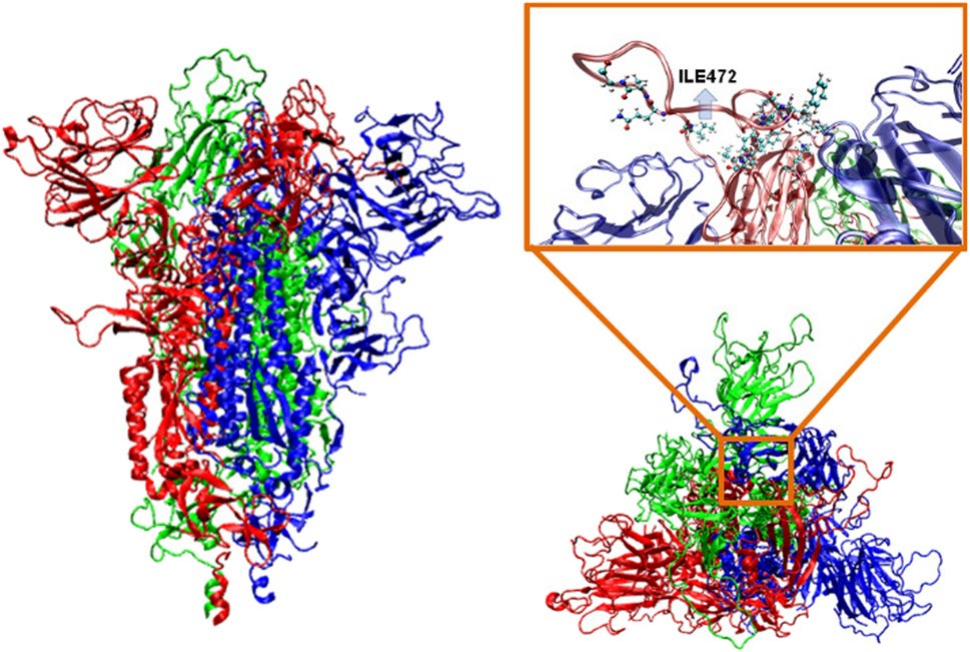
Lateral and frontal views of the SARS-CoV-2 homo-trimeric spike protein. The subset image (in the box) at the right indicates with balls the residues that are crucial for ACE2 binding

Initially, tentative classical molecular dynamics (MD) simulations were performed considering the SARS-CoV-2 spike homo-trimeric glycoprotein [22], as shown in Fig. 1. This was introduced in a previously equilibrated box with 212,198 water molecules and 440 ·OH radicals explicitly defined. The whole system was described at the molecular mechanics (MM) level using the Amber force field [25]. After a production MM-MD trajectory of 250 ns at 310 K using the procedure described in the Methods section, the preferred ·OH···residue close contacts was estimated.

**Fig. 2:**
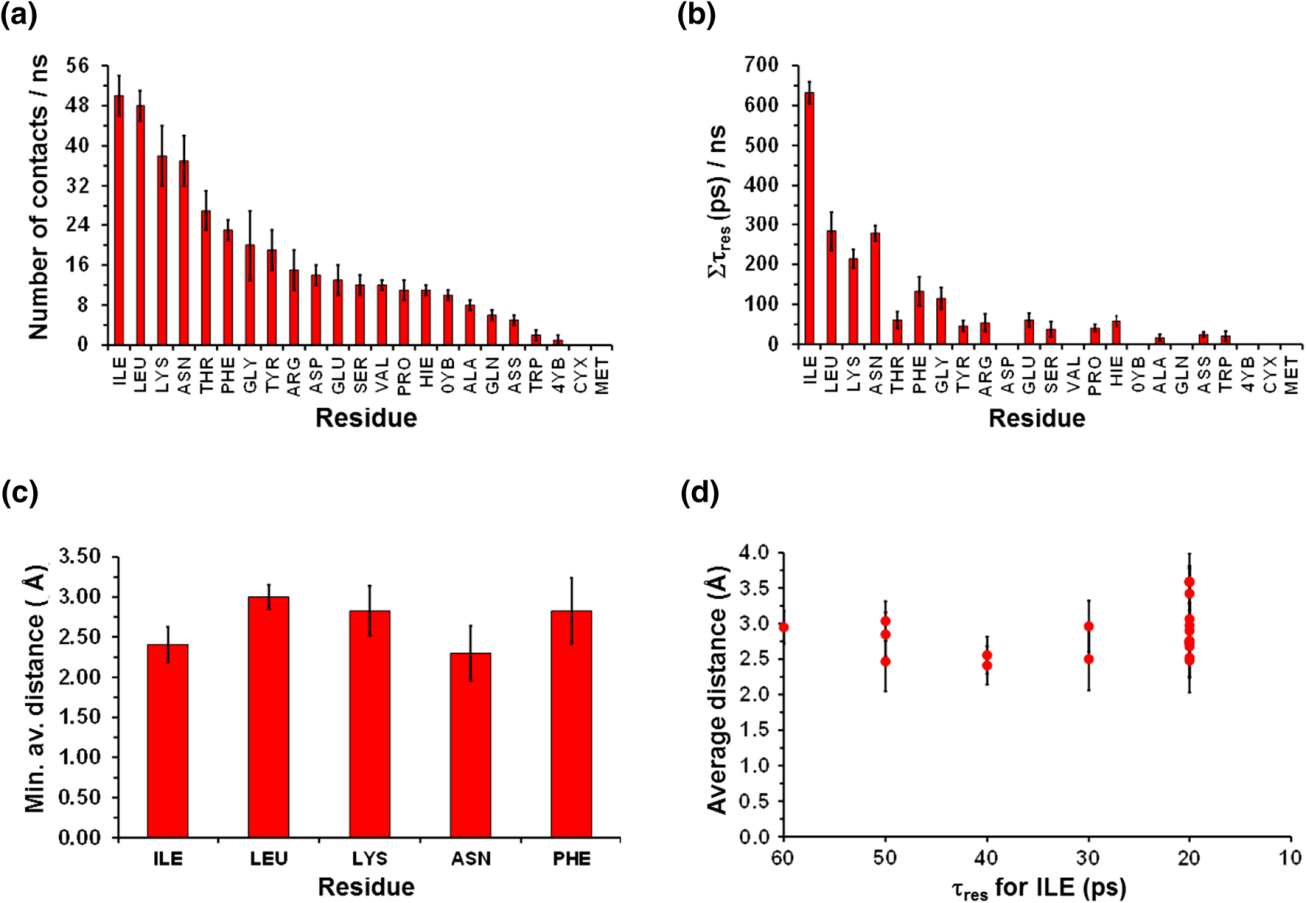
Results from classical MD simulations involving the SARS-CoV-2 homo-trimeric spike protein and the ·OH radical: **a** Number of contacts between the different residues of the protein and each radical type (cutoff distance < 4 Å); **b** Accumulated contact time between the residues and the protein residues (only residence times longer than 2 ps have been considered); **c** Minimum averaged contact distance between the five residues with higher accumulated constant time and the ·OH radical; **d** Averaged contact distance between ·OH radical and the ILE residue as a function of the residence time

As the life time of the ·OH···residue contacts was found to be very short (≤ 80 ps), analyses were performed in blocks of 1 ns (i.e., the ·OH half-life time) for the last 150 ns of the trajectory (*i.e.,* once the complete equilibration of the system was ensured), and the values averaged. The highest number of close contacts with ·OH, which were defined using a threshold distance of 4.0 Å, was obtained for the L-Isoleucine (ILE) residue, followed by L-Leucine (LEU) and L-Lysine (LYS) (Fig. 2a). These results are consistent with those of an early study where the reaction of the ·OH with the aliphatic C − H bonds of amino acids was studied by ^2^H NMR [40]. It was found that ^1^H/^2^H exchange by ·H abstraction occurred in the following descending order: LEU > ILE > VAL > ARG > LYS > TYR > …. The residence time (*τ*_res_) of each contact ranged from 80 to 10 ps, independently of the residue. Representation of the accumulated *τ*_res_ along one ns (Σ*τ*_res_) for the different residues (considering only *τ*_res_ ≥ 20 ps) indicated that ILE exhibited the longest contact (Fig. 2b), suggesting that such residue may play a crucial role in modification processes driven by radicals. Interestingly, analysis of the distance between the two closest atoms in the contacts with *τ*_res_ ≥ 20 ps reveals slightly shorter values for ILE and ASN than for other residues. This is also shown when the average distance for the closest contact is compared, as is depicted for the five residues with the highest Σ*τ*_res_ (Fig. 2c). Moreover, analysis of the average distance for contacts between ·OH and ILE residues with *τ*_res_ ≥ 20 ps revealed values systematically short, independently of Σ*τ*_res_ (Fig. 2d).

Chemical reactions related to ·OH attack to the residues of viral proteins are relevant to understand the influence of the oxidative stress on modification and/or degradation processes [41]. Modifications in glycoproteins can cause an increase in viral infectivity in primates and humans [42]. For example, ILE at position 544 augments the infectivity of Ebola virus (EBOV) and Reston virus (RESTV), regardless of host species, which was attributed to a more pronounced viral fusion activity [43]. Among coronavirus modifications, earlier findings suggested that a single-point mutation at the RBD, which is a core region that binds the cellular receptor human angiotensin-converting enzyme 2 (ACE2; see Fig. 1) and mediates the fusion of the viral and cellular membranes, could affect the recognition process [42].

Focusing on ILE, ·OH converts the residue into a radical by ·H abstraction. Early studies performed using ^2^H NMR spectra on amino acids showed that ILE, LEU and VAL behave similarly [40]. The oxidative modification of such aliphatic amino acids, when are not integrated in the sequence of proteins, depends on their radical stability and, as a consequence, the preferred abstraction occurs at the branch point (C^β^–H for ILE, C^γ^–H for LEU and C^β^–H for VAL). For such reasons, to investigate how the amino acids inserted in the protein structure are affected by the radical species merits attention because the reaction ability of those radicals can affect the binding region stability.

Considering the tendency of ·OH to be attracted by ILE in the spike of SARS-CoV-2, the radical-mediated deprotonation reaction of such residue was examined by means of a hybrid QM/MM–MD scheme using M06-2X Density functional. This technique has been previously used in large biological systems [44]. For this purpose, ILE residue showing the highest amount of close contacts with ^·^OH in classical MM-MD trajectories was selected for hybrid QM/MM-MD simulations. This corresponds to ILE472, which is located at the RBD [45]. Indeed, the enhanced transmissibility of SARS-CoV-2 UK variant has been related to changes within RBD of the spike [46].

**Fig. 3:**
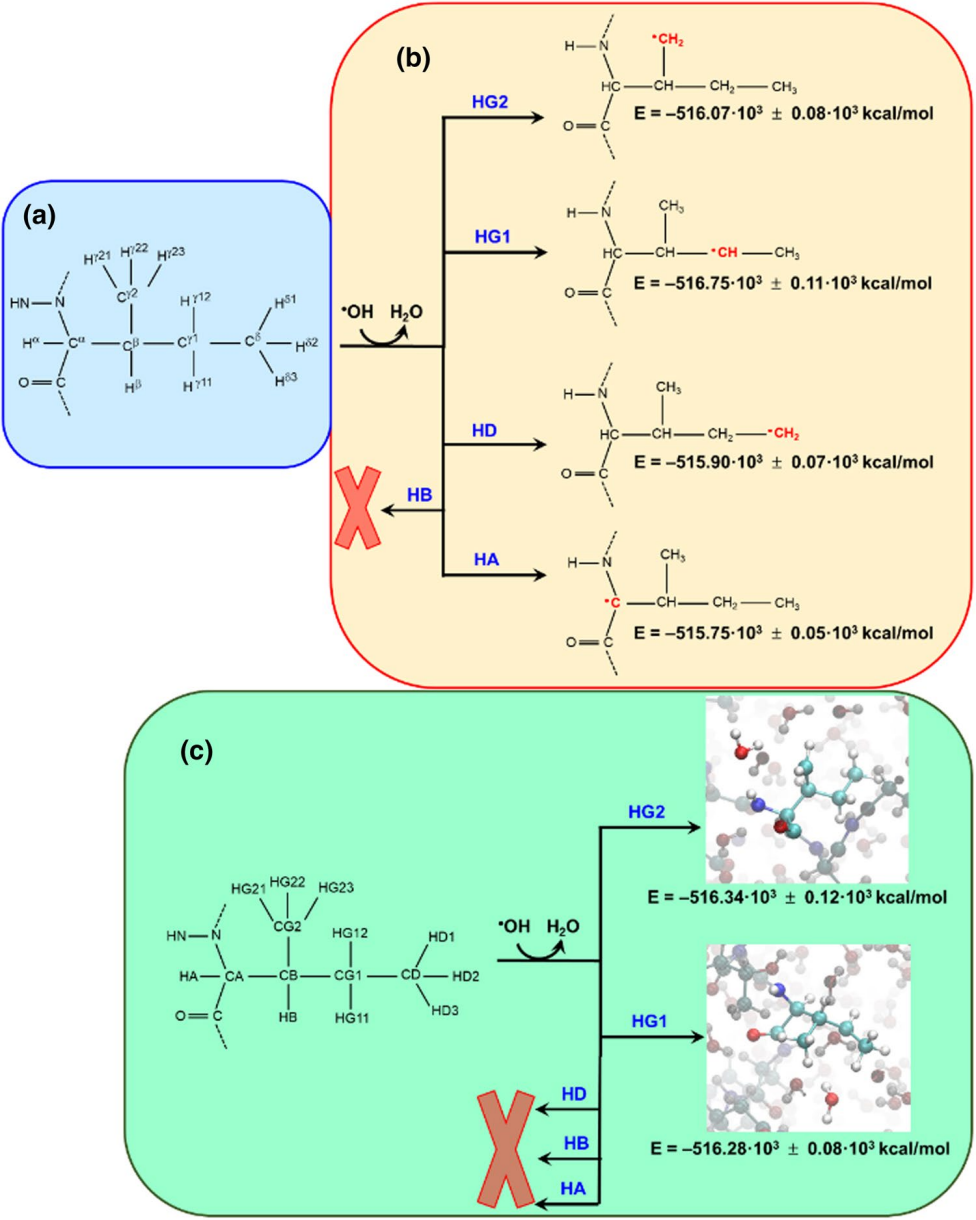
Schemes displaying **a** atoms labeling for ILE and **b**, **c** Successful ^·^H abstraction processes obtained using MQ/MM-MD simulations when a (C–)H···^·^OH distance of b 1.4 Å or **c** 1.7 Å was initially imposed

For QM/MM-MD simulations, an ·OH radical close to the ILE472 residue, together with the last and first atom of the adjacent residues (i.e., the carbonyl carbon atom of GLU471 and the peptidic nitrogen atom of TYR473), was treated at the QM level (active zones). The rest of the system, which consisted on the RBD region, 103 ions (53 Cl^–^ and 50 Na^+^) and 15,928 water molecules remained described classically. Ten representative snapshots, with one ^·^OH radical close (< 3.5 Å) to each of the ten hydrogen atoms of the ILE residue (*i.e.,* H^α^, H^β^, H^γ11^, H^γ12^, H^γ21^, H^γ22^, Hγ^23^, H^δ1^, H^δ2^ and H^δ3^ in Fig. 3a), were randomly chosen among the last 30 ns of the classical MD simulation. As ·H abstraction is the first step in the ·OH-mediated oxidation of aliphatic amino acids [47], for the active zone of each selective snapshot the oxygen atom of ·OH radical was initially approached 1.4 Å from the corresponding hydrogen atom of ILE472. After equilibration using the protocol described in the ESI, QM/MM-MD simulations were run for 0.5 ps.

After 30–60 fs, ^·^H abstraction was observed for all the hydrogens with exception of H^β^ (Fig. 3b). Analysis of the total energy for the resulting systems (listed in Fig. 3b) revealed that the order of stability of the products from ·OH-mediated abstraction is ranked as follows: H^γ1^ > H^γ2^ > H^δ^ > H^α^. However, the QM energy of the active zone was practically identical for all systems, suggesting that the stability of the products is mainly defined by the surrounding protein environment. Although earlier experimental studies evidenced that the abstraction of an ·H atom from the Gly residue usually occurs at the C–H^α^ bond [48, 49], a more recent ab initio study on a LEU dipeptide model (*i.e.,* NH_2_COCHRNHCHO with R = CH_2_CH(CH_3_)_2_) proved the importance of steric interactions in ·H abstraction [41]. Authors did not find a clear distinction among C^β^–H, C^γ^–H and C^δ^–H sites while the reactivity of the C^α^–H bond was lower, which was attributed to steric effects and the tendency of the ·OH to form hydrogen bonds with NH and C = O groups [41]. More recently, ab initio and DFT calculations on a quite similar dipeptide model but with less hydrogen bonding capacity at the N-terminus (*i.e.,* CH_3_CONHCHRCOOH with R = (CH_2_)_4_CH_3_) revealed favored ·H abstractions for the C^α^–H and C^ε^–H bonds [50].

In a very recent work, Gross and coworkers [51] used ^18^O isotopic labeling to study the ·H abstraction from LEU and ILE C–H bonds belonging to short and, probably, non-structured peptides. These authors found that the preferred abstraction occurred at the branch point (i.e., C^γ^–H and C^β^–H for LEU and ILE, respectively), which was attributed to the classical stability ranking of aliphatic radicals (tertiary > secondary > primary). Instead, the ·OH-mediated ·H abstraction of ILE, as amino acid, followed by ^2^H NMR detection of ^1^H/^2^H induced exchange indicated that the C^γ2^–H and C^δ^–H sites are the most reactive [40]. Overall, the results allow us to suggest that the discrepancies among the different studies in the most reactive site come from the flexibility of the alkyl side chain of ILE and the ability of the radical to interact with the surrounding chemical environment. Furthermore, it depends on the flexibility or rigidity of the backbone that, in turn, is influenced by the secondary and tertiary structure of the peptide / protein.

Theoretical QM calculations on model dipeptides [41, 50] showed that the characteristic distances in the C·····H····OH transition state associated with the ·H abstraction reaction are around 1.2 (C····^·^H) and 1.3 Å (H····O). This feature indicates that the reactivity predicted by QM/MM-MD simulations for the different C–H bonds of the ILE residue can be overestimated since the initially imposed (C–)H····OH distance, 1.4 Å, could led to a drastic and artificial decrease of the barrier for the ^·^H transfer. In order to reduce this effect, QM/MM-MD simulations on the SARS-CoV-2 protein were repeated but increasing the initial (C–)H···^·^OH distance to 1.7 Å. This value is large enough to provide a more realistic description of the reactivity, which is defined by the probability of contact with the right spatial orientation between the C–H and ^·^OH groups. QM/MM-MD simulations were performed considering three different cases for each of the ten hydrogen atoms of the ILE residue. Accordingly, a total of 30 QM/MM-MD trajectories, 0.5 ps long each one, were run. The results are summarized in Fig. 3c.

A total five trajectories resulted in a successful ^·^H abstraction, which represents ~ 17% of the run simulations. No ·H abstraction was achieved for the C^α^–H, C^β^–H and C^δ^–H sites, whereas three and two abstractions occurred at the C^γ1^–H and C^γ2^–H bonds, respectively. This feature suggests that the energy barriers for the ^·^H^γ1^ and ·H^γ2^ abstractions are lower than for the other hydrogens, which has been attributed to the higher accessibility of the ·OH radicals to such atoms when ILE forms part of the complex supramolecular structure of the SARS-CoV-2 spike protein. Figure 3c indicates that the γ2-radical product (γ2-·ILE472) is more stable than the γ1-radical one (γ1-^·^ILE472), even though the energy difference found among the products coming from the same reaction site confirms the large influence of the surrounding environment on this ^·^H abstraction process.

**Fig. 4:**
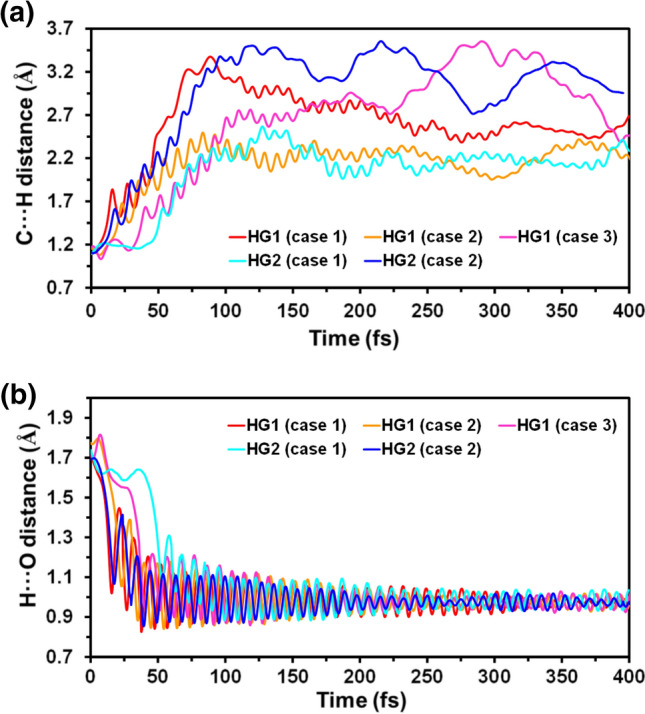
Temporal evolution of the **a** C···H and **b** H···O distances involved in the conversion of ILE and ^·^OH to ^·^ILE and water, respectively, as derived from QM/MM-MD simulations

Figure 4a-b shows the temporal evolution of the C···H distance, which is associated with the formation of the radical of ILE472 (·ILE472) through the breaking of the C–H bond, and the H···O distance involved in the conversion of ·OH to water. As is shown, during the first 50 fs of the trajectories, the C···H distance increases from 1.1 to ~ 1.8 Å while the distance O···H decreases from 1.7 Å to ~ 1.0 Å. After this period, the vibration of the O–H bond is progressively dampened, while the distance C···H of the broken C–H bond increases as the water molecule moves away from the reaction site.

Although the capture of the ·H from C–H bonds when ·OH interacts with the protein was expected, our results allow to conclude that such atom is not extracted from the protein backbone, which would result in the cleavage of the peptide backbone. Instead, a different reaction pathway occurs for the obtained ·ILE472, in which ^·^H abstraction from exposed amino acid side chains facilitates the protein modification by reaction with ROS in physiological environments. In this work we have analyzed the reaction of ·ILE472 from the RBD with both the very reactive ·OH and the nitric oxide radical (·NO), which is produced by NO synthases and is able to modify biomolecules such as proteins, particularly in RNA virus [52]. Two different 200 fs long QM/MM-MD trajectories were run for each ·ILE472 (γ1- and γ2-radical products) and each ROS (·OH and ·NO). For this purpose, the radical was initially approached to a distance of 1.6 and 2.0 Å of the reactive site.

**Fig. 5:**
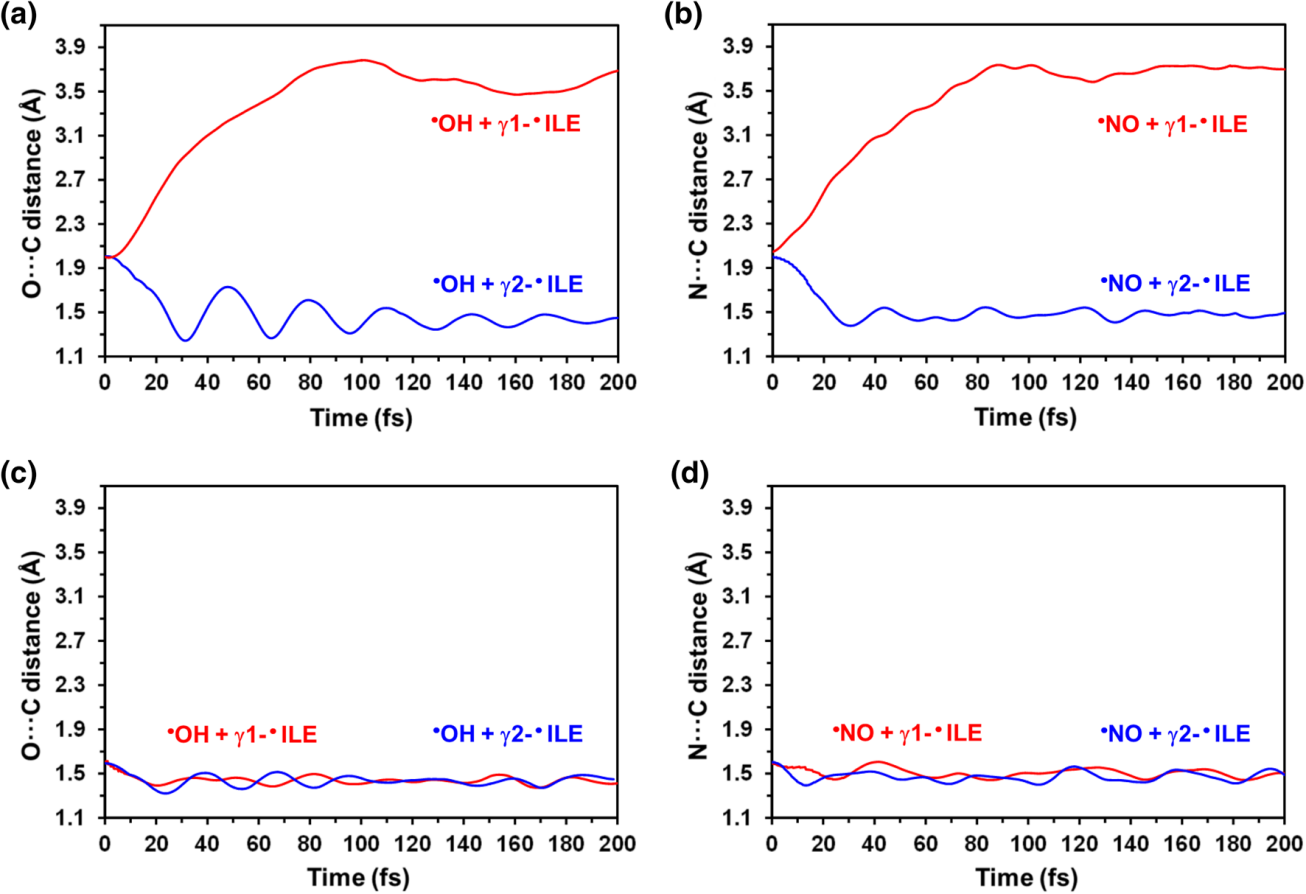
Variation of the **a**, **c** O···C and (b, d) N···O distance as obtained from QM/MM-MD simulations for the following hydroxylation and nitrosylation reactions: **a**, **c** ^·^OH + γ1-^·^ILE472 and ^·^OH + γ2-^·^ILE472; and **b**, **d** ^·^NO + γ1-^·^ILE472 and ^·^NO + γ2-^·^ILE472. The initial inter-radical distance was of **a**, **b** 2.0 Å and **c**, **d** 1.6 Å

The addition of the two radicals to γ1-·ILE472 was unsuccessful when the initial distance was of 2.0 Å (Fig. 5a-b). Thus, O···C and N···C distances grew from 2.0 Å to 3.54 ± 0.05 Å and 3.69 ± 0.04 Å (averaged over the last 50 fs of trajectory), respectively. Conversely, when the barrier associated with the addition process was artificially reduced by decreasing the initial inter-radical distance to 1.6 Å, both the γ1-hydroxy-ILE472 and γ1-nitro-ILE472 were successfully formed. The resulting average O–C and N–C bond distances were 1.43 ± 0.03 and 1.51 ± 0.03 Å, respectively (Fig. 5c-d). Interestingly, the hydroxylation and nitrosylation of γ2-·ILE472 was successful in all cases, independently of initial inter-radical distance. Moreover, the obtained O–C and N–C distances were practically identical, independently of the initial inter-radical distance (O–C: 1.42 ± 0.03 Å and N–C: 1.469 ± 0.03), as is shown in Fig. 5.

**Fig. 6:**
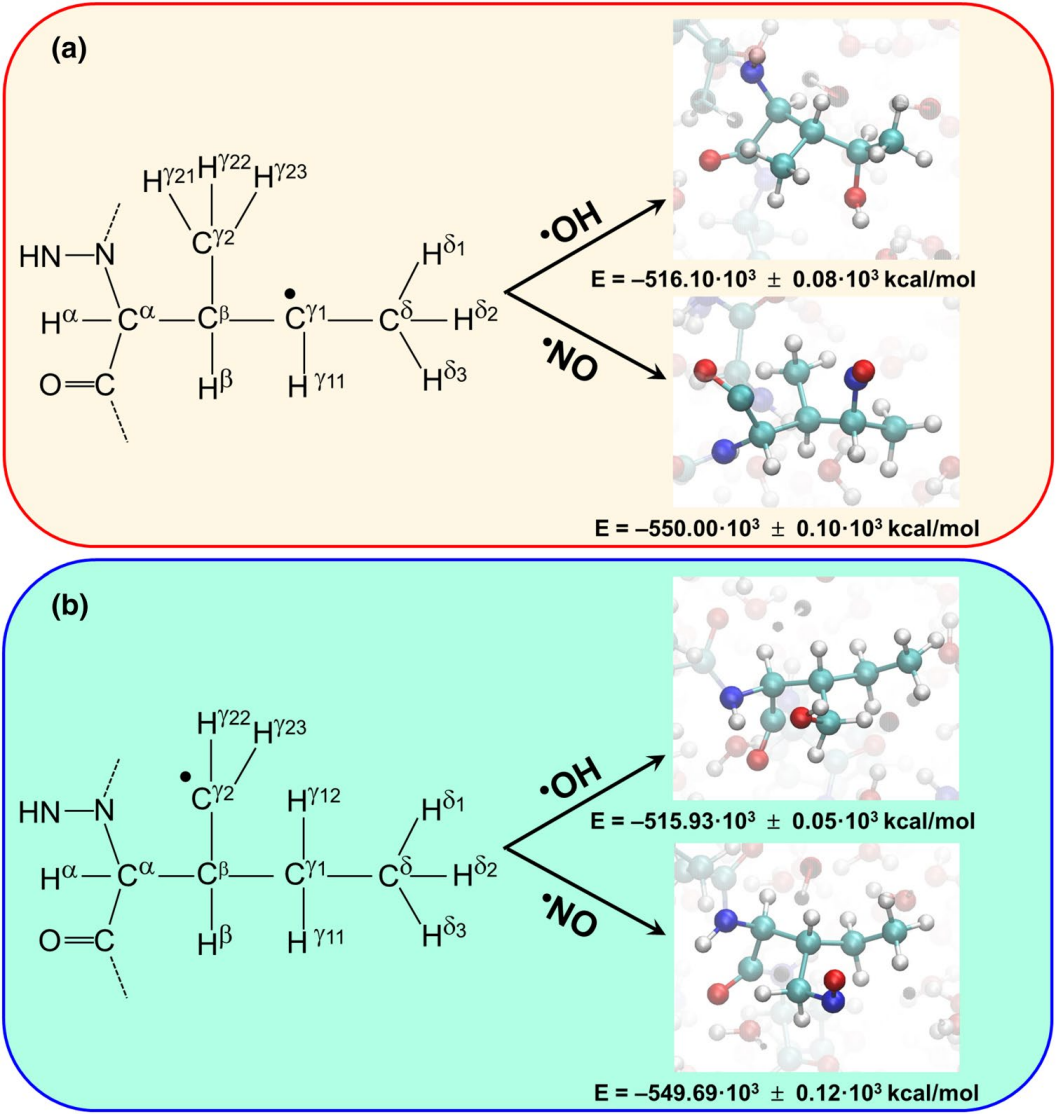
Scheme displaying the results from the reactions of the two ^·^ILE472 radicals with the ^·^OH and NO^·^ species as obtained from QM/MM-MD simulations: **a** γ1-^·^ILE472 and **b** γ2-^·^ILE472. The product from each reaction is displayed using a solid sphere model for modified residue. The energy of each product is expressed as the average values for the last 50 fs ± the standard deviation

**Fig. 7:**
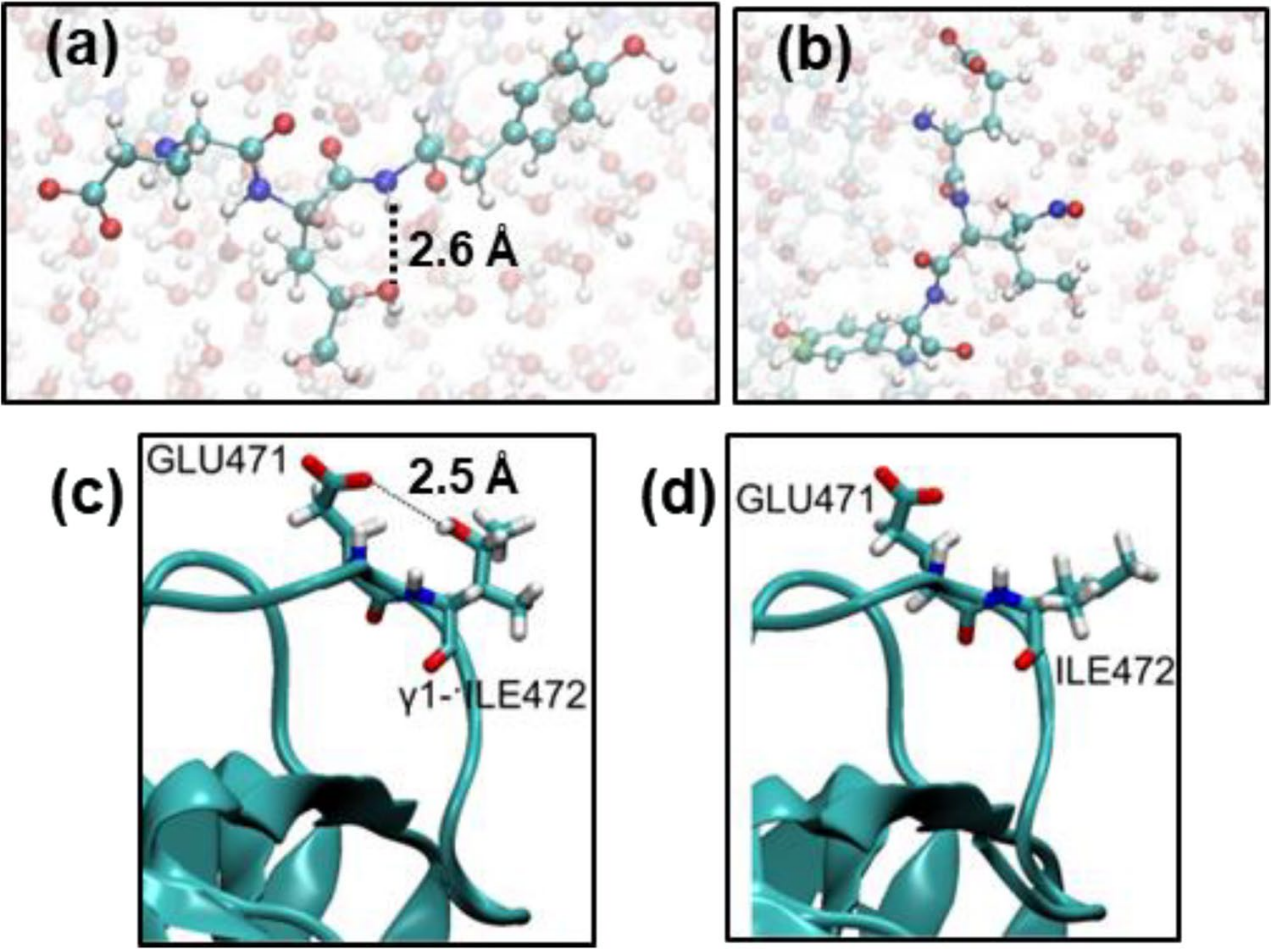
**a** Intramolecular hydrogen interaction between the backbone of TYR473 and the hydroxyl group of γ1-hydroxy-ILE472. **b** The intramolecular hydrogen interaction between displayed in (**a**) was not found for γ2-nitro-ILE472. **b** Intramolecular interaction between the carboxylate of GLU471 and the γ1-hydroxy-ILE472 residue sporadically formed at the end of the QM/MM-MD trajectory. **d** Intramolecular hydrogen bonding interactions were not possible in the wild type protein, in which the ILE472 was not mutated

Figure 6 shows the energies of the products obtained from the hydroxylation and nitrosylation of the ^·^ILE472 residue and the variation of the O···C and N···C distances, respectively. As it can be seen, the systems containing γ1-hydroxy- and γ1-nitro-ILE472 are significantly favored with respect to those obtained from the γ2-^·^ILE472 reaction. Although this feature is consistent with the fact that γ2-^·^ILE472 is more stable than γ1-^·^ILE472, the stability of the γ1-hydroxy-ILE product with respect to γ2-nitro-ILE has been attributed to the formation of a favorable N–H···O hydrogen bond (Fig. 7a), which is absent in the latter (Fig. 7b).

It is worth noting that the effect of amino acid modifications at the RBD region of the spike protein may affect the protein–protein recognition process [53]. For example, in addition of the interaction with the backbone of TYR473 (Fig. 7a), the γ1-hydroxy-ILE472 residue occasionally interacts with the side group of GLU471 (Fig. 7c) along the MM/QM-MD trajectory. These interactions, which were not detected in the wild type (Fig. 7d), may alter the recognition process of ACE2. Indeed, the incidence of ROS-induced modifications is expected to increase with the relevance of the residue in the recognition mechanism.

**Fig. 8:**
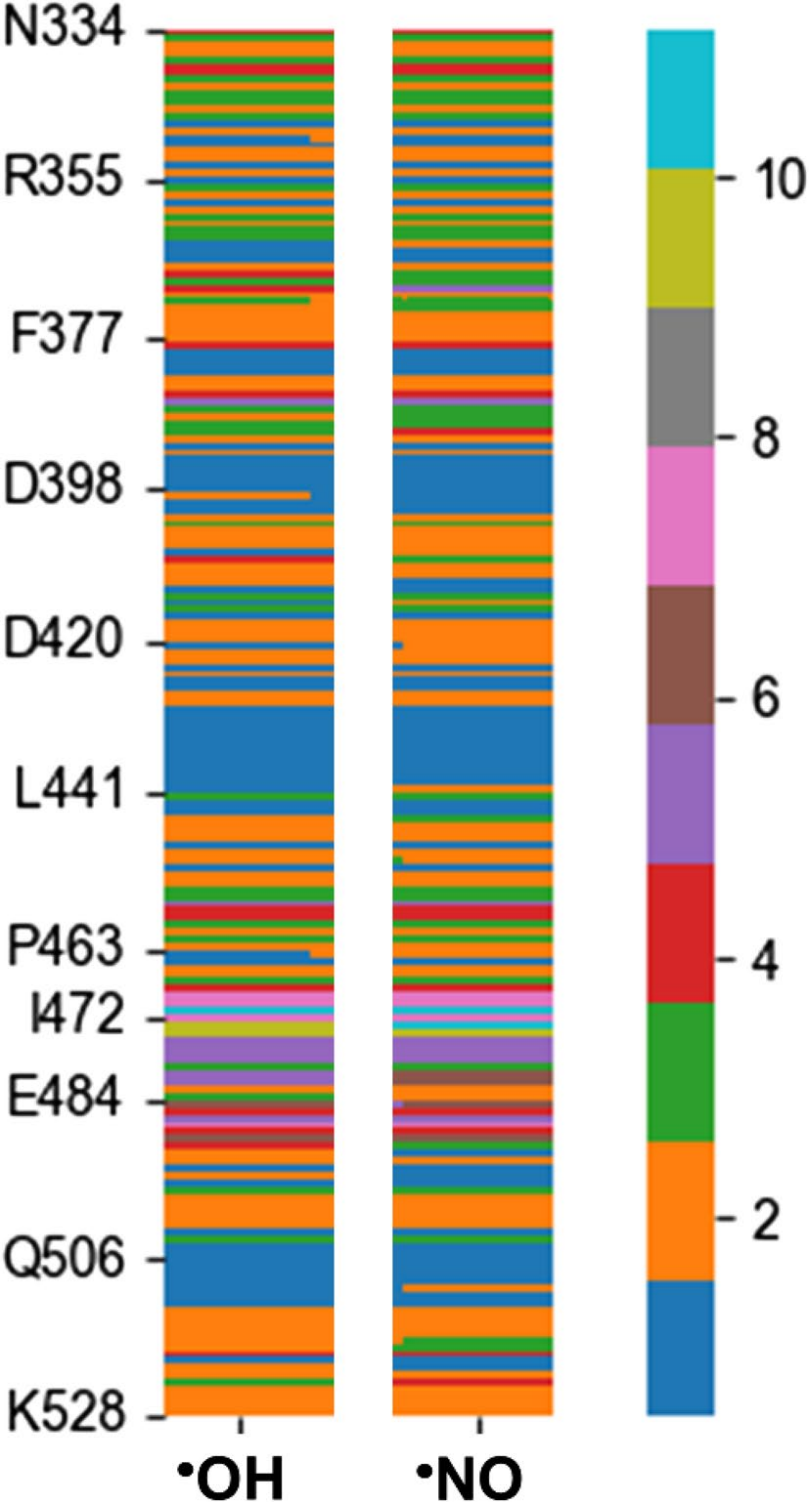
Heat-map of RMSD values calculated for all atoms with respect to the crystal for the RBD of γ1-hydroxy-ILE472 (^·^OH) and γ2-nitro-ILE472 (^·^NO)

Figure 8, which displays the heat-map of RMSD values calculated for all atoms with respect to the crystal for the RBD of γ1-hydroxy-ILE472 and γ2-nitro-ILE472, indicates that some regions, including the ILE472 residue, undergo major structural alterations. In particular, although many tracts exhibit RMSD values ≤ 3 Å, which is reasonably low considering the dynamics of both the backbone and the side chins, the RMSD increases up to values of 9 Å for residues surrounding ILE472. This feature suggests that modifications induced by oxidative stress can play a crucial process in the recognition of ACE2.

## Conclusions

In summary, the consequences of oxidative stress in the SARS-CoV-2 spike glycoprotein have been modeled in terms of ^·^OH-mediated ^·^H abstraction, and the subsequent hydroxylation or nitrosylation of the resulting radical. The results show that ILE residues are accessible and interact with ^·^OH. The hydrogen abstraction is more favored at the γ1- and γ2-positions of the eight ILE residues located at the RBD. Furthermore, formed γ1-^·^ILE and γ1-^·^ILE radicals react with ^·^OH and NO^·^ species, the hydrogen bonding pattern of the resulting modifications being different from that found for the wild type protein. Multi-scale simulations reveal not only the implications of the oxidative stress in the stability of the RBD but also show that the recognition pattern of the modified residues may change. All in all, in silico results evidence the necessity of experimental studies based on NMR and mass spectrometry to locate and quantify the formation of radicals and modifications in in the SARS-CoV-2 spike glycoprotein.
